# Comprehensive machine learning boosts structure-based virtual screening for PARP1 inhibitors

**DOI:** 10.1093/bib/bbaf631.064

**Published:** 2025-12-12

**Authors:** Klaudia Caba, Viet-Khoa Tran-Nguyen, Taufiq Rahman, Pedro J Ballester

**Affiliations:** Department of Bioengineering, Imperial College London, United Kingdom; Unité de Biologie Fonctionnelle et Adaptative, Université Paris Cité, France; Department of Pharmacology, University of Cambridge, United Kingdom; Department of Bioengineering, Imperial College London, United Kingdom

## Abstract

**Aims:**

Developing and evaluating target-specific machine learning (ML) scoring functions (SFs) to enhance structure-based virtual screening for Poly (ADP-ribose) polymerase 1 (PARP1), a key DNA repair enzyme and validated target in BRCA-mutated cancers. Existing PARP1 inhibitors face limitations in selectivity and toxicity, motivating ML-driven strategies for identifying improved chemotypes [1].

**Methods:**

Bioactivity data from ChEMBL were combined with property-matched decoys generated using DeepCoy. Ligands were docked into the PARP1 crystal structure with Smina, and training and test sets were prepared. Five ML algorithms, random forest, extreme gradient boosting, support vector machines (SVM), and neural network models, were trained in regression and classification modes using multiple featurisation techniques, including protein-ligand extended connectivity (PLEC) fingerprints, GRID, and Morgan fingerprints, as described in our recent publication [2]. Models were benchmarked against generic SFs using enrichment factor (EF1%), normalised EF1% (NEF1%) and precision-recall curves.

**Results and conclusions:**

Target-specific ML SFs consistently surpassed generic approaches, with a SVM regressor trained on PLEC fingerprints achieving the best performance (EF1% = 38.8, NEF1% = 0.764). Models retained strong predictive ability on a structurally dissimilar test set, confirming generalisation and recovery of diverse inhibitors. These results demonstrate that ML-tailored SFs substantially improve virtual screening for PARP1 and provide an effective route toward novel, selective anticancer compounds.

**References:**

1. Franzese E., Centonze S., Diana A., Carlino F., Guerrera LP., Di Napoli M., et al. ‘PARP inhibitors in ovarian cancer.’ Cancer Treatment Reviews. 2019;73:1–9.

2. Caba K., Tran-Nguyen VK., Rahman T., Ballester P.J. ‘Comprehensive machine learning boosts structure-based virtual screening for PARP1 inhibitors.’ J Cheminform. 2024;16.

